# Influence of social isolation caused by coronavirus disease 2019 (COVID-19) on the psychological characteristics of hospitalized schizophrenia patients: a case-control study

**DOI:** 10.1038/s41398-020-01098-5

**Published:** 2020-11-24

**Authors:** Jun Ma, Tingting Hua, Kuan Zeng, Baoliang Zhong, Gang Wang, Xuebing Liu

**Affiliations:** 1 Affiliated Wuhan Mental Health Center, Tongji Medical College of Huazhong University of Science & Technology, Wuhan, China; 2 Department of Psychiatry, Henan Mental Hospital, The Second Affiliated Hospital of Xinxiang Medical University, Xinxiang, China

**Keywords:** Psychiatric disorders, Scientific community

## Abstract

Coronavirus disease 2019 (COVID-19) has been classified as a pandemic, and mental hospitals located in epidemic centers have been affected. Social isolation is an important and irreplaceable measure to control the spread of the epidemic. In this study, schizophrenic patients who were subjected to social isolation after close contact with COVID-19 patients were used as participants to explore the impact of social isolation on common inflammatory indicators and psychological characteristics. A total of 30 patients with schizophrenia were recruited from Wuhan Mental Health Center. In addition, 30 ordinary schizophrenic patients were matched with the isolation group and were recruited from another branch of Wuhan Mental Health Center as controls. We compared the differences in common inflammatory indicators and psychological characteristics between the isolated group and the control group, and longitudinal comparison of the differences in the above indicators before and after isolation among the isolation group. The Chinese Perceived Stress Scale (CPSS) score, Hamilton Depression Scale (HAMD) score and Hamilton Anxiety Scale (HAMA) score of the isolation group were significantly higher than those of the control group (*p* = 0.00, 0.00, 0.00, respectively). The C-reactive protein (CRP) level, CPSS score, HAMA score and Pittsburgh sleep quality index (PSQI) score of the isolation group were significantly higher after isolation (*p* = 0.01, 0.00, 0.00, 0.00, 0.00, respectively). Inpatients of schizophrenia suffered from social isolation due to COVID-19 have a severe psychological burden. Social isolation caused patients to develop a weak inflammatory state and led to worse anxiety and sleep quality.

## Introduction

Coronavirus disease 2019 (COVID-19) was an acute respiratory infectious disease caused by a novel coronavirus (SARS-CoV-2)^1^, and this disease has been classified as a global pandemic by the World Health Organization (WHO). Psychiatric specialist hospitals in China treat many mental patients who are homeless, have poor self-care abilities, and require long-term hospitalization; hospitals that are located near the epicenter of the epidemic have been severely impacted by the pandemic^2,3^. Most countries around the world control the spread of the epidemic by implementing social distancing measures and isolating infected persons. Until vaccines and specific antibodies are developed, the isolation of suspected and confirmed patients may be the most effective and practical means of preventing the spread of the disease^4^.

As early as 1988, *House* and his colleagues published a landmark prospective epidemiological review of social isolation on human health^5^. It was particularly surprising that social isolation was also an important risk factor for morbidity and mortality, similar to smoking, obesity, a sedentary lifestyle, and high blood pressure^5^. Social isolation is a powerful source of stress both for animals and humans^6,7^. Researchers have suggested that social isolation-induced stress in rats exhibits similar signs and symptoms as human mental illnesses (such as anxiety, depression, and schizophrenia)^8,9^. A systematic review found relatively consistent evidence that social isolation was associated with the deterioration of mental health^10^. Two other systematic reviews found that there is an association between social networks and depression, such that having rich, large, and high-quality social relationships is beneficial for preventing depression^11,12^. In line with the findings from those studies, two reports targeting special populations, such as adolescents^13^ and pregnant women^14^, also indicated that social isolation had a negative impact on the psychological health outcomes of study participants.

Strictly speaking, compared with the general population, the social networks of long-term hospitalized psychiatric patients with severe disorders are very narrow and limited; to a certain extent, they are already in a state of social isolation. However, previous studies of humans under social isolation did not use isolation in the same way as it is currently being used to prevent the spread of COVID-19. Therefore, we examined hospitalized schizophrenic patients who had close contact with COVID-19 patients and were subjected to social isolation to explore the influence of social isolation on the psychological characteristics of hospitalized schizophrenic patients.

## Materials and methods

### Participants

The 30 participants we recruited were schizophrenic patients who under long-term hospitalization at Wuhan Mental Health Center. These patients were medically isolated from 10 January 2020 to 30 April 2020, due to having close contact with COVID-19 patients. The inclusion criteria were as follows: (1) close contact with COVID-19 patients; (2) after isolation and screening, COVID-19 infection was finally excluded; (3) medical isolation time ≥ 14 days; (4) diagnosed with schizophrenia in accordance with the Diagnostic and Statistical Manual of Mental Diagnostic criteria (DSM-VI); (5) hospitalization time before isolation ≥2 years; (6) gender is not limited; and (7) aged between 20 and 70 years old. During the study, we excluded patients with bipolar disorder, material dependence, mental disorders, personality disorders, dementia, and other serious cognitive disorders caused by physical diseases as well as patients with serious physical diseases and other infectious diseases during medical isolation.

In addition, we recruited 30 uninfected schizophrenic patients who were matched with the isolation group in terms of age, gender, education experience, course of the disease, and length of stay in another branch of Wuhan Mental Health Center as controls.

This study was approved by the ethics committee of the Wuhan Mental Health Center. All patients or their guardians provided written informed consent.

### Study design and procedures

This study was designed as a case-control study. We compared the differences in common inflammatory indexes and psychological characteristics between isolated patients in the epicenter of the epidemic and unquarantined patients. Furthermore, we analyzed the differences in the above indicators among the isolation group over time (i.e., before and after isolation).

On 30 January 2020, one of the hospital districts of Wuhan Mental Health Center officially established isolation wards to isolate confirmed and suspected COVID-19 patients and individuals who had close contact with those patients. The isolation ward was designed to hold 1–3 persons/room. Patients from wards with COVID-19-confirmed patients who needed to be quarantined usually underwent the following tests: routine blood tests, C-reactive protein (CRP) tests, chest CT scans, and SARS-CoV-2 nucleic acid testing. The investigators recruited patients who met the inclusion criteria, who had close contact with COVID-19 patients in the same ward, and who were required to be isolated and observed. After patients were transferred to the isolation ward, their daily life and general activities were limited. In addition to brief communications during doctors’ rounds and daily work by the nursing staff, some patients isolated to a single room usually had no people to communicate with, and the interpersonal communication activities among patients who were isolated in a non-single room were limited to patients in the same ward.

Before these close contacts were transferred to the isolation ward, we evaluated the severity of their psychotic symptoms, depression symptoms, anxiety symptoms, psychological stress, and sleep quality. The inflammatory indexes of the same period were recorded, including leukocytes, neutrophils, lymphocytes, and CRP, which were recorded simultaneously. When these patients had been quarantined for 14 days and tested negative for COVID-19, they were transferred out of the isolation ward. We repeated the evaluations on the 10th-14th days of isolation. As of 30 April 2020, a total of 30 patients had been released from isolation and are included in this study.

At the peak of the Chinese epidemic in February and March 2020^15^, the control group was long-term inpatients with schizophrenia from another branch of Wuhan Mental Health Center, where had implemented closed management since the beginning of the epidemic. The epidemic was relatively contained in this hospital. While the hospital was closed, family visits were forbidden, new patients were not admitted, fixed medical staff was not mobile, and other measures were implemented to prevent the spread of the disease. The daily activities of patients did not change. Patients could visit other adjacent wards, participate in activities at certain times, eat together, queue up to take medicine, watch TV together, do radio gymnastics, etc. More importantly, these patients did not encounter other patients who were confirmed or suspected to be infected with COVID-19, and furthermore, they did not come in contact with medical personnel wearing protective clothing.

At the peak of the epidemic, the same assessments and laboratory tests that were administered to the isolation group were administered to these patients.

### Instruments

The electronic medical record system was used to extract the common inflammatory indexes and general clinical data of the isolation group and control group. The Chinese Perceived Stress Scale (CPSS) was used to assess the severity of participants’ psychological stress, the Positive and Negative Symptom Scale (PANSS) was used to assess the severity of participants’ psychiatric symptoms, the Hamilton Depression Scale (HAMD) was used to assess the severity of participants’ depressive symptoms, the Hamilton Anxiety Scale (HAMA) was used to assess participants’ anxiety symptoms, and the Pittsburgh sleep quality index (PSQI) was used to assess participants’ sleep quality.

The assessment of control group was performed by one psychiatrist, and the assessment of the isolation group was completed by a deputy chief psychiatrist and a psychiatrist. To ensure the consistency of the evaluation results, all psychiatrists participating in the evaluation underwent the same training.

### Statistical analysis

The normally distributed continuous data are expressed as the mean and standard deviation (SD), and categorical data are expressed as frequencies. Independent sample *t*-tests and paired *t*-tests were performed to compare continuous variables with normal distributions, and chi-square tests were performed to compare categorical variables. The significance level of all the statistical tests was set as *p* < 0.05 (two-tailed). Data analysis was performed using IBM SPSS (SPSS, Inc., Chicago, IL, USA) version 26.0.

## Results

### Differences between the general clinical characteristics of the isolation group and control group

There was no significant difference between the isolation group and the control group in terms of gender, age, duration of psychiatric illness, length of hospitalization, and years of education (*p* = 0.60, 0.50, 0.93, 0.68, 0.93, respectively) (Table 1).

**Table 1:** Differences between the general clinical characteristics of the isolation group and control group.

	Isolation group (*n* = 30)	Control group (*n* = 30)	*χ*^2^/*t*	*p*
Gender (female vs male)	18 vs 12	15 vs 15	0.61	0.60
Age	43.17 ± 11.55	45.00 ± 9.16	−0.68	0.50
Course of psychosis (years)	20.20 ± 9.26	20.00 ± 8.42	0.88	0.93
Length of stay (years)	4.90 ± 2.67	5.17 ± 2.35	−0.41	0.68
Years of education (years)	10.30 ± 3.43	10.37 ± 3.07	−0.79	0.93

### Differences in common inflammatory indexes and general psychological characteristics between the isolation group and control group

There were no statistically significant differences in the common inflammatory indicators between the isolation group and the control group, including white blood cell count, neutrophil count, lymphocyte count, and CRP (*p* = 0.13, 0.19, 0.30, 0.59, respectively). The CPSS score, HAMD score, and HAMA score of the study group were significantly higher than those of the control group (*p* = 0.00, 0.00, 0.00, respectively); however, the PANSS score and PSQI score were not significantly different between the two groups (*p* = 0.52, 0.32, respectively) (Table 2).

**Table 2:** Differences in common inflammatory indexes and general psychological characteristics between isolation group and control group.

	Isolation group (*n* = 30)	Control group (*n* = 30)	*t*	*p*
Leukocyte count	5.61 ± 1.70	6.25 ± 1.53	−1.53	0.13
Neutrophil count	3.49 ± 1.33	3.94 ± 1.36	−1.32	0.19
Lymphocyte count	1.58 ± 0.71	1.77 ± 0.68	−1.05	0.30
CRP	7.03 ± 4.17	6.49 ± 3.45	0.54	0.59
CPSS	24.97 ± 5.39	11.60 ± 3.98	12.94	0.00*
PANSS	90.23 ± 7.75	88.20 ± 15.52	0.64	0.52
HDMD	19.97 ± 1.47	15.90 ± 3.49	5.91	0.00*
HDMA	15.47 ± 1.48	12.77 ± 3.01	4.40	0.00*
PSQI	5.57 ± 2.85	4.73 ± 3.56	1.00	0.32

*CRP* C-reactive protein, *CPSS* Chinese Perceived Stress Scale, *PANSS* Positive and Negative Symptom Scale, *HAMD* Hamilton Depression Scale, *HAMA* Hamilton Anxiety Scale, *PSQI* Pittsburgh sleep quality index.

*p < 0.05.

### Differences in common inflammatory indexes and general psychological characteristics in the isolation group before and after social isolation

Compared to before isolation, there were no statistically significant differences in the leukocyte count, neutrophil count, lymphocyte count, PANSS score or HAMD score in the isolation group after isolation (*p* = 0.09, 0.12, 0.13, 0.36, 0.27, respectively). The CRP level, CPSS score, HAMA score, and PSQI score of the isolation group were significantly higher after isolated (*p* = 0.01, 0.00, 0.00, 0.00, 0.00, respectively) (Table 3).

**Table 3:** Differences in common inflammatory indexes and general psychological characteristics in the study group before and after social isolation.

	Before isolation (*n* = 30)	After isolation (*n* = 30)	*t*	*p*
Leukocyte count	5.61 ± 1.70	6.07 ± 1.49	−1.76	0.09
Neutrophil count	3.49 ± 1.33	3.81 ± 1.24	−1.61	0.12
Lymphocyte count	1.58 ± 0.71	1.80 ± 0.76	−1.58	0.13
CRP	7.03 ± 4.17	9.56 ± 3.85	−2.74	0.01*
CPSS	24.97 ± 5.39	27.17 ± 5.25	−3.39	0.00*
PANSS	90.23 ± 7.75	88.27 ± 10.52	0.94	0.36
HDMD	19.97 ± 1.47	20.83 ± 3.82	−1.14	0.27
HDMA	15.47 ± 1.48	16.97 ± 2.04	−3.89	0.00*
PSQI	5.57 ± 2.85	7.50 ± 3.50	−3.00	0.00*

*CRP* C-reactive protein, *CPSS* Chinese Perceived Stress Scale, *PANSS* Positive and Negative Symptom Scale, *HAMD* Hamilton Depression Scale, *HAMA* Hamilton Anxiety Scale, *PSQI* Pittsburgh sleep quality index.

*p < 0.05.

## Discussion

To the best of our knowledge, this was the first study to explore the impact of social isolation caused by COVID-19 on the psychological status of inpatients with schizophrenia. The results of this study indicate that inpatients suffered from social isolation due to COVID-19 have higher levels of psychological stress and more severe anxiety and depression than those in hospitals that are not suffered from. After social isolation, inpatients with schizophrenia showed higher levels of CRP and psychological stress, more severe anxiety, and worse sleep quality.

Social isolation is a composite concept that encompasses the quantity, structure, and quality of social networks and emotional resources for the appraisal of relationships^16^. However, the social isolation examined in our research is relatively simple—specifically, we examined short-term severe physical isolation. The purpose of this isolation was to minimize the spread of COVID-19 in our hospital. As a form of stress, social isolation is related to mental illness. Social isolation promotes oxidative stress and hypothalamic–pituitary–adrenal (HPA) axis activation and weakens the expression of genes that control inflammation and regulate the glucocorticoid response^17^. This process is the potential biological mechanism of many kinds of mental diseases, including anxiety disorder, depression disorder, and schizophrenia^18^. Summarizing the above results and combining them with our research results reveal that social isolation is a negative factor in the evolution of the mental illness. In other words, this process leads to higher levels of psychological stress, more anxiety and more depression.

Angela and colleagues showed that adult prairie voles exhibit depression-like behavior after being isolated for 4 weeks^19^. They also found that isolation increased the anxiety-like behavior of prairie voles^20^. However, it was unclear whether social isolation selectively increased anxiety-like behavior instead of depression^21^. A study of elderly people in the United States found that social discontinuity predicts more severe symptoms of depression and anxiety^22^. The findings of two other studies on special populations in Japan, namely, people without social support and perinatal women, found that social isolation led to higher levels of anxiety^23^. In this study, participants showed higher stress levels, more serious anxiety levels, and worse sleep quality after nearly 2 weeks of physical isolation. These results were not completely consistent with previous research results. As mentioned earlier, the isolation caused by COVID-19 is unprecedented. The isolation studied herein was acute, physical, specific, and mechanical rather than chronic, cultural, abstract, and flexible. These might be the biggest differences from previous studies involving people in social isolation. In addition, the worries caused by the epidemic, and the changes in the hospitalization environments (e.g., sick friends suddenly disappearing due to being isolated, and medical staff wearing protective gear rather than ordinary work clothes) also adversely affected the mental state of participants. These were confounding factors in this study, but the study was more complex.

During our research, all the inflammatory indicators were within the normal range. In addition to the significant increase in CRP levels, although the inflammation indicators examined in this study did not differ significantly after isolation, they all increased. Some studies suggest that the inflammatory system might be activated after social isolation to enhance the response to pressure and to avoid the threat of injury^24,25^. Interestingly, positive social interactions and good social support were associated with reduced levels of inflammation^26^. A meta-analysis showed that social isolation was associated with increased levels of CRP^26^. Busch et al.^27^ indicated that people with larger social networks showed lower levels of CRP and a lower leukocyte count; in contrast, greater social pressure is associated with higher levels of inflammation. The results of these studies provide a foundation for the conclusion of this study: social isolation is a special form of inflammation.

This study also has certain limitations. Due to the limitations of the research conditions, the impacts of a wide range of inflammatory markers, such as interleukin, interferon gamma, and tumor necrosis factor, were not examined with respect to acute social isolation, and we did not measure the correlation between these inflammatory indicators and changes in mental state. In addition, the short research duration and the small sample size also had adverse effects on our research results.

In short, the increased psychological burden on hospitalized schizophrenic patients caused by COVID-19-induced social isolation led to a weak inflammatory state, increased anxiety and decreased sleep quality.
